# Supplementary material for: PKC isoforms activate LRRK1 kinase by phosphorylating conserved residues (Ser1064, Ser1074 and Thr1075) within the CORB GTPase domain
Source: Biochem J. 2022 Sep 23;479(18):1941–65. doi: 10.1042/BCJ20220308 (PMC9555798; doi:10.1042/BCJ20220308)
Supplement: Supplementary Material [file BCJ-479-1941-s1.pdf]

Supplementary Section

PKC isoforms activate LRRK1 kinase by phosphorylating conserved residues (Ser1064, Ser1074 and Thr1075) within the COR_B GTPase domain

Asad U Malik[1,4*], Athanasios Karapetsas[1*], Raja S. Nirujogi[1,4], Deep Chatterjee[2,4], Toan K. Phung[1,4], Melanie Wightman[1], Robert Gourlay[1], Nick Morrice[3], Sebastian Mathea[2,4], Stefan Knapp[2,4] and Dario R Alessi*[1,4]

* Authors made an equal contribution to the Study

1 Medical Research Council (MRC) Protein Phosphorylation and Ubiquitylation Unit, School of Life Sciences, University of Dundee, Dow Street, Dundee DD1 5EH, United Kingdom.

2 Structural Genomics Consortium, Buchmann Institute for Molecular Life Sciences and Institute for Pharmaceutical Chemistry, Johann Wolfgang Goethe-University, Max-von-Laue-Str. 9, D-60438 Frankfurt am Main, Germany

3 AB Sciex, Alderley Park, Macclesfield SK10 4TG, United Kingdom

4 Aligning Science Across Parkinson's (ASAP) Collaborative Research Network, Chevy Chase, MD, 20815.

*Correspondence to Dario R Alessi (d.r.alessi@dundee.ac.uk)

SFigure 1: Quantification of time course of PMA treatment in HEK293 Flp-In T-Rex cells. Quantified immunoblotting data from Figure 1C are presented as ratios of pRab7ASer72/total Rab7A and total PKCα/tubulin (mean ± SEM).

SFigure 2: PKCα stoichiometrically phosphorylates LRRK1. The indicated concentrations of PKCα (0-100 nM) was incubated with recombinant wild type (WT) insect cell expressed recombinant (r) LRRK1[20-2015] (50 nM) in the presence of Mg[γ-^{32}P]ATP (500 cpm/pmol). Each sample is analysed in quadruplicate. Reactions were terminated after 30 min with SDS-sample buffer. 80% of each reaction was resolved by SDS-polyacrylamide electrophoresis, stained by Coomassie blue (upper panel), and subjected to autoradiography (middle panel). Dried bands were then excised from the gel and counts per minute were evaluated using a scintillation counter to determine stoichiometry of LRRK1 phosphorylation. Data is presented mean ± SEM.

SFigure 3. Time-course and dose-dependence of activation of recombinant insect LRRK1 by PKCα. (A) Step-1 of the LRRK1 kinase activation assay was setup with insect cell produced recombinant wild type (WT) LRRK1[20-2015], the indicated concentrations of PKCα, ± PKC inhibitor GÖ6983 (10 µM) in the presence of non-radioactive MgATP for 30 min. PKC phosphorylated LRRK1 was then diluted 1.5-fold into a kinase assay containing recombinant Rab7A (1 mM) in the presence of non-radioactive MgATP (Step 2). Reactions were terminated after 30 min with SDS-sample buffer and subjected to a multiplexed

immunoblot analysis using the LI-COR Odyssey CLx Western Blot imaging system with the indicated antibodies. Combined immunoblotting data from 2 independent biological replicates (each performed in duplicate) are shown. Lower panel quantified immunoblotting data are presented as ratios of pRab7ASer72/total Rab7A (mean ± SEM) relative to levels observed with no PKCα added (give a value of 1.0). (**B**) As in (**A**) except in step 1 of the kinase activity assay 100 nM PKCα was incubated with immunoprecipitated GFP-LRRK1 for the times indicated.

SFigure 4: Recombinant PKC Alpha further activates PMA-stimulated LRRK1. HEK293 Flp-In T-REx cells stably expressing wild type (WT) GFP-LRRK1 were treated ± 1 μg/ml doxycycline for 24 h to induce GFP-LRRK1 expression. Cells were serum-starved for 16 h and stimulated ± 160 nM phorbol 12-myristate 13-acetate (PMA) for 30 min and lysed. GFP-LRRK1 immunoprecipitated and aliquoted as indicated. Each aliquot contains GFP-LRRK1 immunoprecipitated from 1 mg of HEK293 cell lysate. Step-1 of the LRRK1 kinase activation assay was setup by incubating GFP-LRRK1 aliquots with PKCα (100 nM) in the presence of non-radioactive MgATP for 30 min. PKC phosphorylated LRRK1 was then diluted 1.5-fold into a kinase assay containing recombinant Rab7A (1 mM) in the presence of non-radioactive MgATP (Step 2 of the kinase assay). Reactions were terminated after 30 min with SDS-sample buffer analysed by multiplexed immunoblot analysis using the LI-COR Odyssey CLx Western Blot imaging system with the indicated antibodies (upper panel). Combined immunoblotting data from 2 independent biological replicates (each performed in duplicate) are shown. Lower panel quantified immunoblotting data are presented as ratios of pRab7ASer72/total Rab7A (mean ± SEM) relative to levels observed with no PKCα added (given a value of 1.0).

SFigure 5. Heatmap of phosphorylation sites identified via HCD analysis.
Kinase inactive LRRK1[D1409A, 20-2015] (200 nM) was incubated ± PKCα (400 nM) the presence of MgATP. Reactions were terminated after 30 min with SDS-sample buffer and reactions resolved by SDS-polyacrylamide electrophoresis and gel stained by Coomassie blue. The gel bands containing LRRK1 were digested with mixture of AspN, Chymotrypsin and Trypsin+Lys-C. The resultant peptides were analyzed by HCD fragmentation acquired on Orbitrap Exploris 240 MS platform. The high confidence phosphosites that are identified and analyzed using MaxQuant are depicted as a heatmap representing the relative abundance of identified phosphosites versus protease utilized ± PKCα. The grey color denotes no phosphopeptides detected. Side panel show the evolutionary conservation score from 1 (least conserved) to 9 (most conserved) of each of the identified phosphorylation sites determined calculated the Consurf server ((https://consurf.tau.ac.il/) [52]. Sequences of LRRK1 homologs were obtained from OrthoDB (https://www.orthodb.org/) [53] and alignment of sequences was performed using the MAFFT server (https://www.ebi.ac.uk/Tools/msa/mafft/) [54]. Phosphosites Thr1061 and Ser1064 and their respective conservation scores are highlighted in red.

SFigure 6. Membrane-fraction of LRRK1 colocalizes with plasma membrane marker Na-K ATPase. (Left panel) HEK293 Flp-In T-REx cells stably expressing wild type (WT) GFP-LRRK1 were treated with 1 μg/ml doxycycline for 24 h to induce GFP-LRRK1 expression. Cells were serum-starved for 16 h and stimulated ± 160 nM phorbol 12-myristate 13-acetate (PMA) for 30 min. Cells were permeabilized by liquid nitrogen freeze–thaw to deplete cytosol [48] and

then fixed and stained with mouse anti-Na-K ATPase, chicken anti-GFP and DAPI. **(Right panel)** Co-localization of GFP-LRRK1 and Na-K ATPase was determined from a Mander's coefficient (presented as mean ± SEM) after automatic thresholding. *P* = 0.6425 (ns) by Student's unpaired, two-tailed t test.

SFigure 7. Triple Ser1064, Ser1074 and Thr1075 alanine or glutamate mutations do not affect LRRK1 localization. (Left panel) HEK293 cells were grown on cover slips and transiently transfected with plasmids encoding GFP-LRRK1 wild type (WT) or the indicated mutants of GFP-LRRK1. Cells were serum-starved for 16 h and stimulated ± 160 nM phorbol 12-myristate 13-acetate (PMA) for 30 min. Cells were permeabilized by liquid nitrogen freeze–thaw to deplete cytosol [46] and then fixed and stained with mouse anti-PKCα, chicken anti-GFP and DAPI. (Right panel) Co-localization of GFP-LRRK1 and PKCα, was determined from a Mander's coefficient (presented as mean ± SEM) after automatic thresholding. P > 0.05 (ns) by Student's unpaired, two-tailed t test.

SFigure 1

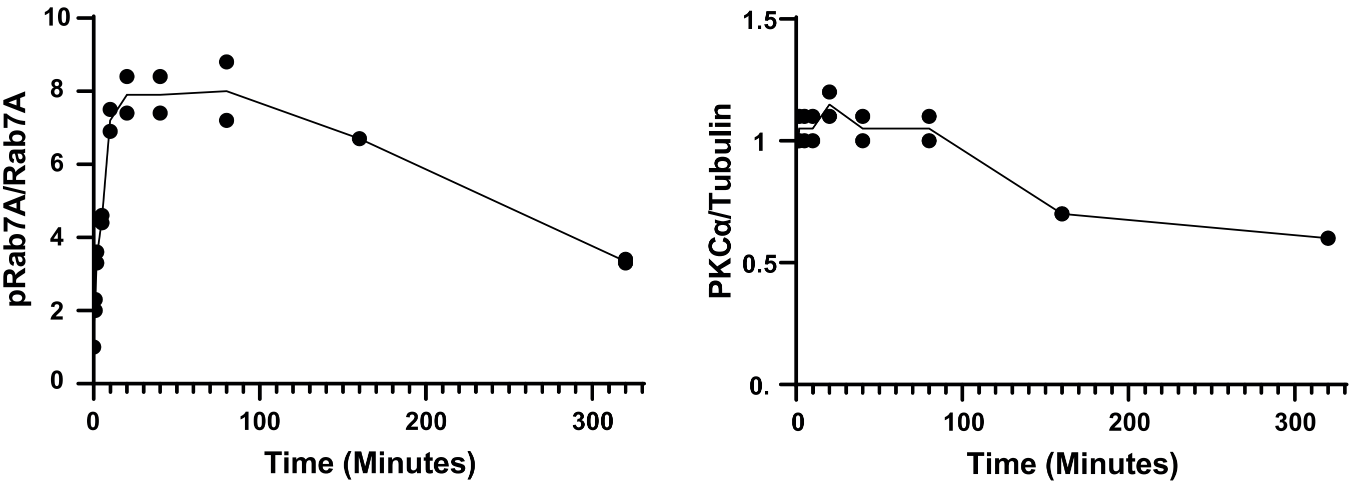

pRab7A/Rab7A
10
8
6
4
2
0
0
100
200
300
Time (Minutes)
PKCα/Tubulin
1.5
1
0.5
0.
0
100
200
300
Time (Minutes)

SFigure 2

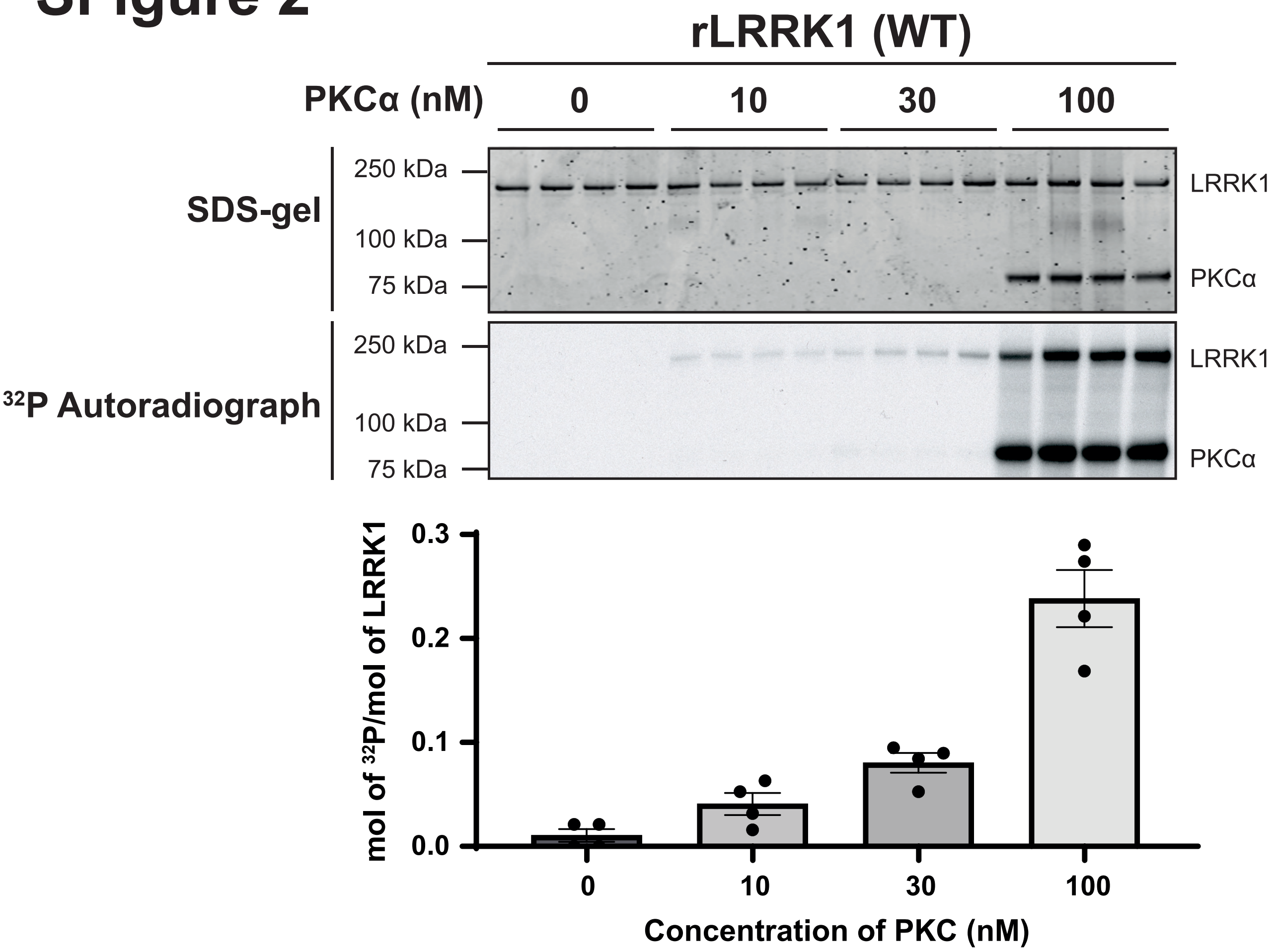

rLRRK1 (WT)
PKCα (nM)
0
10
30
100
SDS-gel
250 kDa
100 kDa
75 kDa
LRRK1
PKCα
^{32}P Autoradiograph
250 kDa
100 kDa
75 kDa
LRRK1
PKCα
mol of ^{32}P/mol of LRRK1
0.3
0.2
0.1
0.0
0
10
30
100
Concentration of PKC (nM)

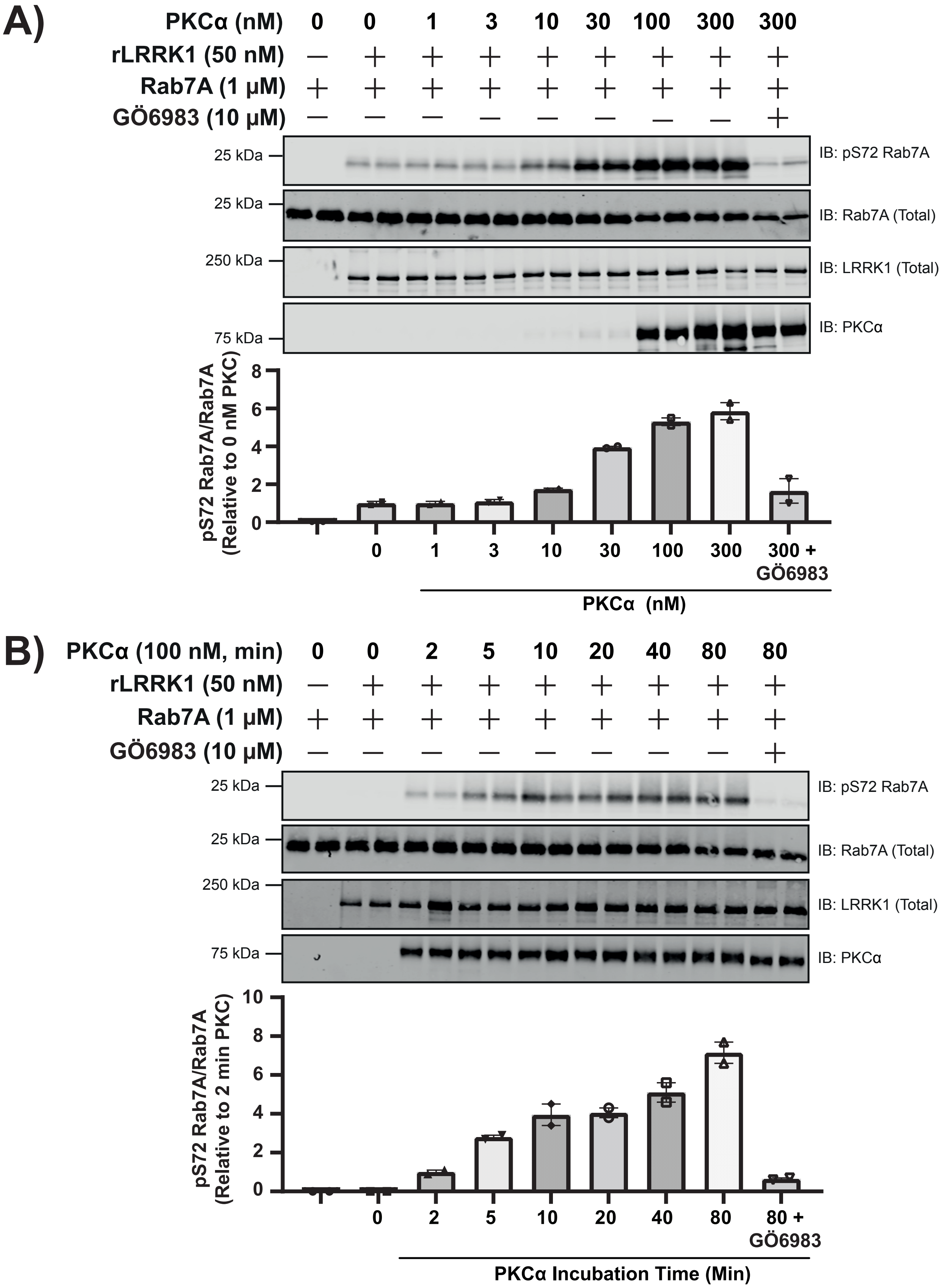

A)
PKCα (nM) 0 0 1 3 10 30 100 300 300
rLRRK1 (50 nM) — + + + + + + + +
Rab7A (1 μM) + + + + + + + + +
GÖ6983 (10 μM) — — — — — — — — +
25 kDa
25 kDa
250 kDa
75 kDa
IB: pS72 Rab7A
IB: Rab7A (Total)
IB: LRRK1 (Total)
IB: PKCα
pS72 Rab7A/Rab7A (Relative to 0 nM PKC)
8
6
4
2
0
0 1 3 10 30 100 300 300 + GÖ6983
PKCα (nM)
B)
PKCα (100 nM, min) 0 0 2 5 10 20 40 80 80
rLRRK1 (50 nM) — + + + + + + + +
Rab7A (1 μM) + + + + + + + + +
GÖ6983 (10 μM) — — — — — — — — +
25 kDa
25 kDa
250 kDa
75 kDa
IB: pS72 Rab7A
IB: Rab7A (Total)
IB: LRRK1 (Total)
IB: PKCα
pS72 Rab7A/Rab7A (Relative to 2 min PKC)
10
8
6
4
2
0
0 2 5 10 20 40 80 80 + GÖ6983
PKCα Incubation Time (Min)

SFigure 3

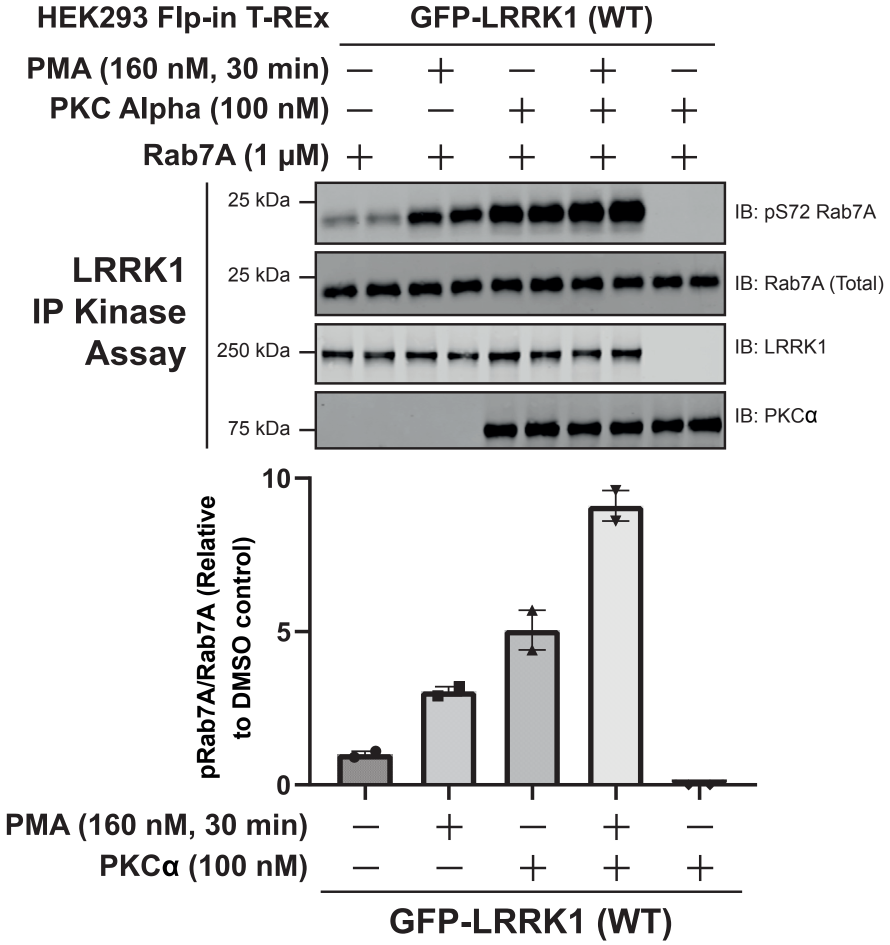

HEK293 Flp-in T-REx
GFP-LRRK1 (WT)
PMA (160 nM, 30 min) — + — + —
PKC Alpha (100 nM) — — + + +
Rab7A (1 μM) + + + + +
LRRK1 IP Kinase Assay
25 kDa
IB: pS72 Rab7A
25 kDa
IB: Rab7A (Total)
250 kDa
IB: LRRK1
75 kDa
IB: PKCα
pRab7A/Rab7A (Relative to DMSO control)
10
5
0
PMA (160 nM, 30 min) — + — + —
PKCα (100 nM) — — + + +
GFP-LRRK1 (WT)

SFigure 4

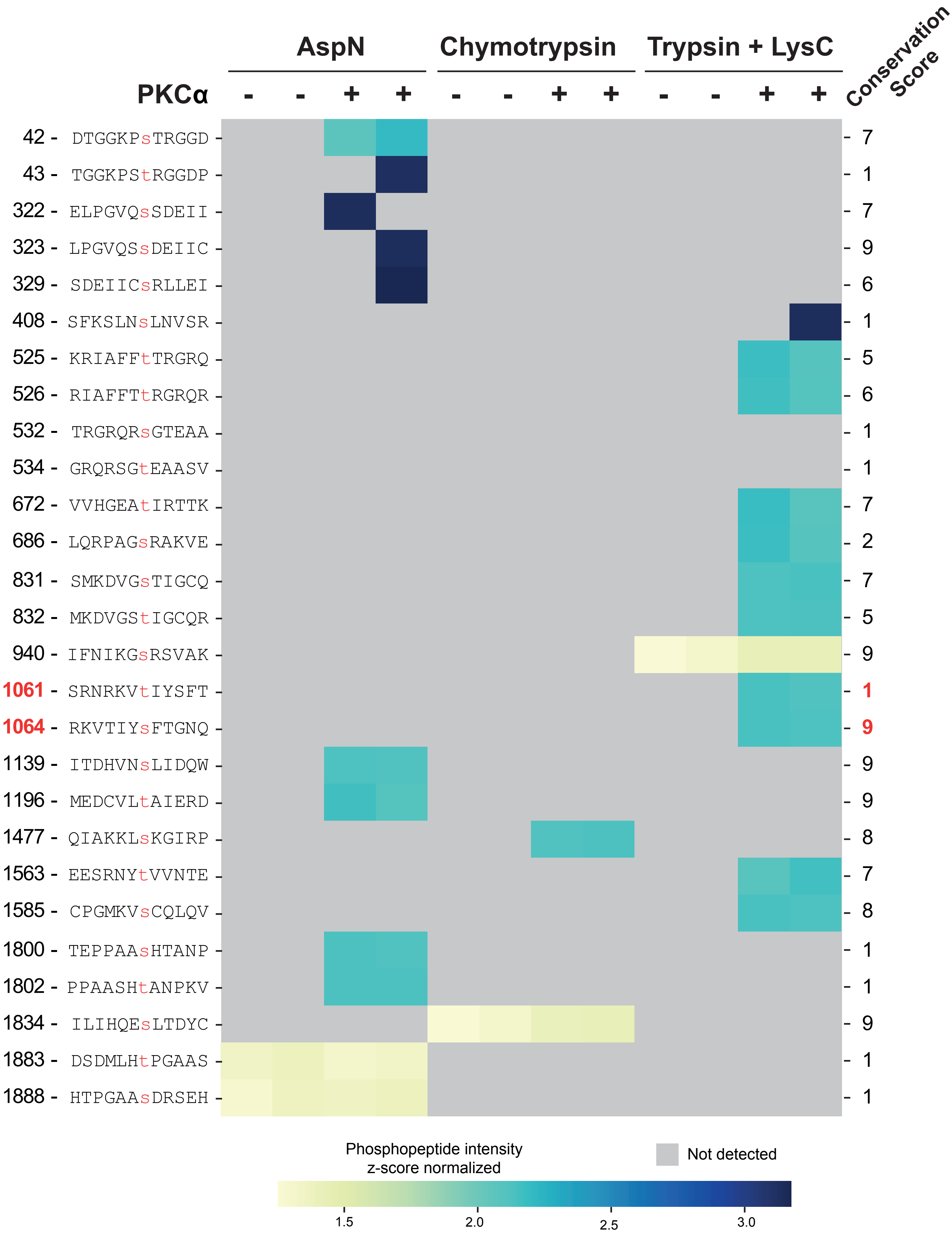
AspN
Chymotrypsin
Trypsin + LysC
Conservation Score
PKCα
- - + + - - + + - - + +
42 - DTGGKPsTRGGD - 7
43 - TGGKPStRGGDP - 1
322 - ELPGVQsSDEII - 7
323 - LPGVQSsDEIIC - 9
329 - SDEIICsRLLEI - 6
408 - SFKSLNsLNVSR - 1
525 - KRIAFFtTRGRQ - 5
526 - RIAFFTtRGRQR - 6
532 - TRGRQRsGTEAA - 1
534 - GRQRSGtEAASV - 1
672 - VVHGEAtIRTTK - 7
686 - LQRPAGsRAKVE - 2
831 - SMKDVGsTIGCQ - 7
832 - MKDVGStIGCQR - 5
940 - IFNIKGsRSVAK - 9
1061 - SRNRKVtIYSFT - 1
1064 - RKVTIYsFTGNQ - 9
1139 - ITDHVNsLIDQW - 9
1196 - MEDCVLtAIERD - 9
1477 - QIAKKLsKGIRP - 8
1563 - EESRNYtVVNTE - 7
1585 - CPGMKVsCQLQV - 8
1800 - TEPPAAsHTANP - 1
1802 - PPAASHtANPKV - 1
1834 - ILIHQEsLTDYC - 9
1883 - DSDMLHtPGAAS - 1
1888 - HTPGAAsDRSEH - 1
Phosphopeptide intensity
z-score normalized
Not detected
1.5
2.0
2.5
3.0

SFigure 5

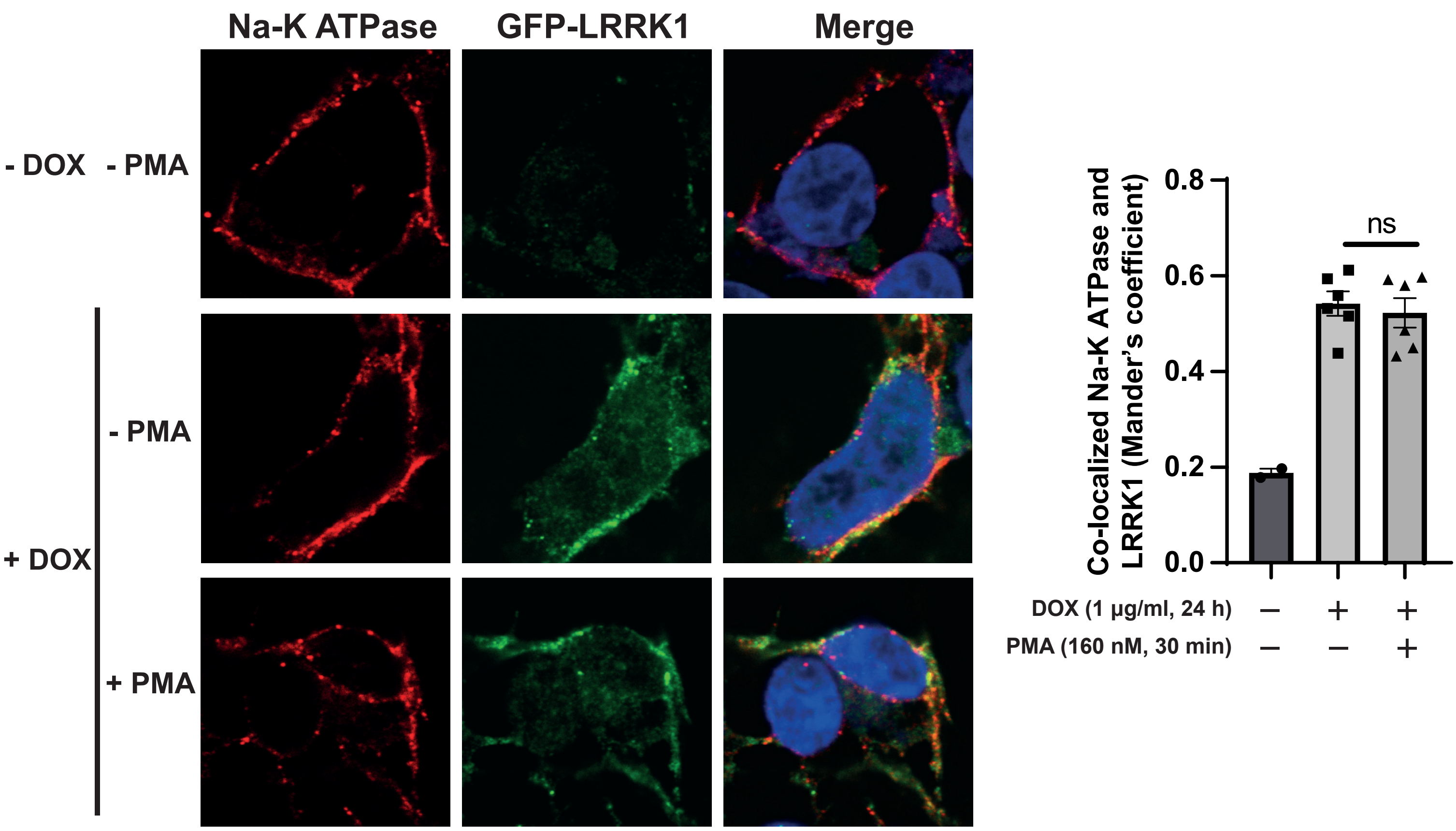
Na-K ATPase
GFP-LRRK1
Merge
- DOX
- PMA
- PMA
+ DOX
+ PMA
Co-localized Na-K ATPase and LRRK1 (Mander's coefficient)
0.8
0.6
0.4
0.2
0.0
ns
DOX (1 µg/ml, 24 h)
PMA (160 nM, 30 min)
–
+
+
–
–
+

SFigure 6

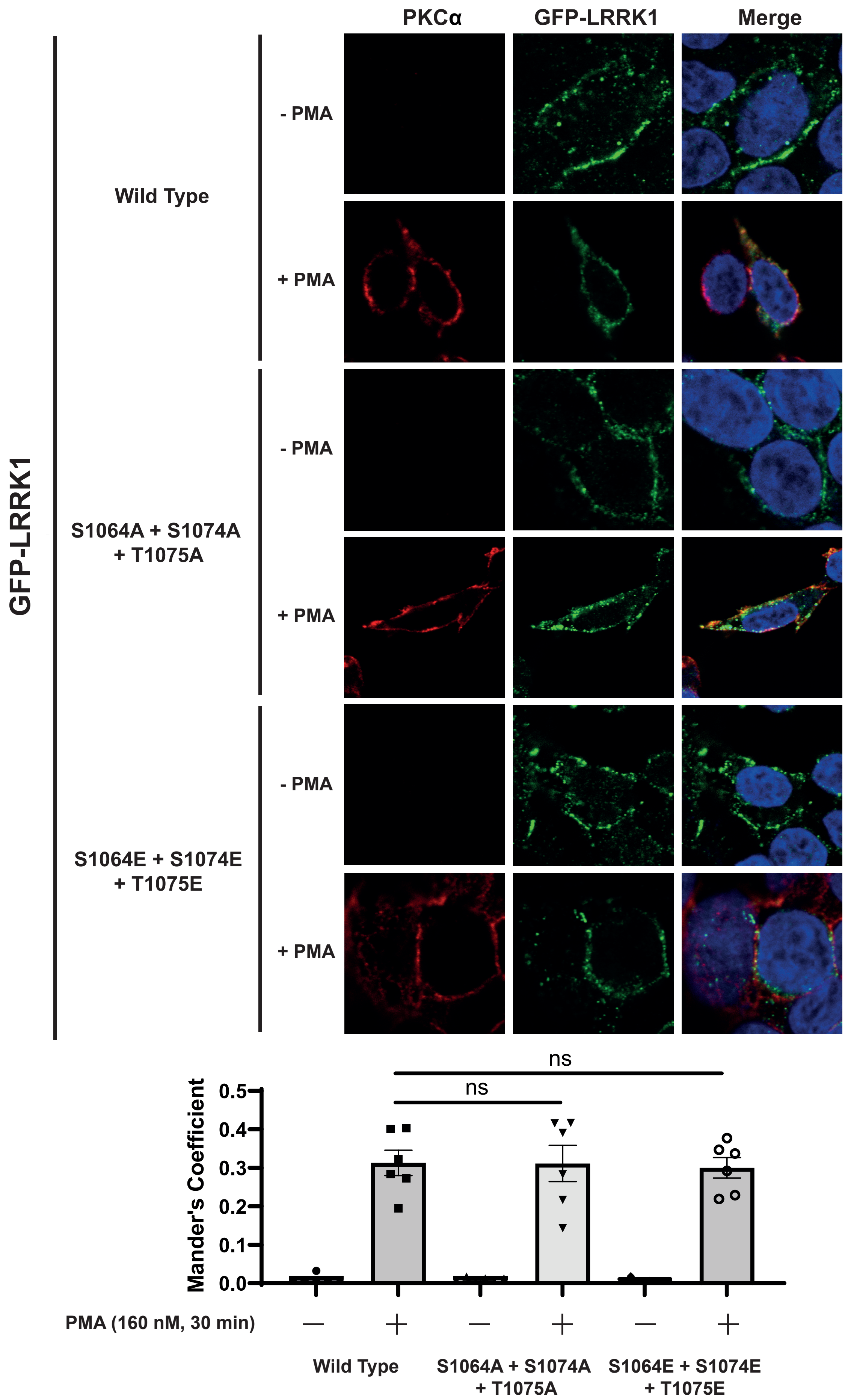
PKCα
GFP-LRRK1
Merge
GFP-LRRK1
Wild Type
- PMA
+ PMA
S1064A + S1074A
+ T1075A
- PMA
+ PMA
S1064E + S1074E
+ T1075E
- PMA
+ PMA
ns
ns
Mander's Coefficient
0.5
0.4
0.3
0.2
0.1
0.0
PMA (160 nM, 30 min)
Wild Type
S1064A + S1074A
+ T1075A
S1064E + S1074E
+ T1075E

SFigure 7
